# Regulatory Role of the RNA N^6^-Methyladenosine Modification in Immunoregulatory Cells and Immune-Related Bone Homeostasis Associated With Rheumatoid Arthritis

**DOI:** 10.3389/fcell.2020.627893

**Published:** 2021-01-21

**Authors:** Danping Fan, Ya Xia, Cheng Lu, Qinbin Ye, Xiaoyu Xi, Qiong Wang, Zheng Wang, Chengyuan Wang, Cheng Xiao

**Affiliations:** ^1^Institute of Clinical Medicine, China-Japan Friendship Hospital, Beijing, China; ^2^Graduate School of Peking Union Medical College, Chinese Academy of Medical Sciences/Peking Union Medical College, Beijing, China; ^3^School of Traditional Chinese Medicine, Beijing University of Chinese Medicine, Beijing, China; ^4^Institute of Basic Research in Clinical Medicine, China Academy of Chinese Medical Sciences, Beijing, China; ^5^Clinical Medical School (China-Japan Friendship Hospital), Beijing University of Chinese Medicine, Beijing, China; ^6^Laboratory for Bone and Joint Diseases, RIKEN Center for Integrative Medical Sciences, Tokyo, Japan; ^7^Department of Plastic Surgery, China-Japan Friendship Hospital, Beijing, China; ^8^Department of Emergency, China-Japan Friendship Hospital, Beijing, China

**Keywords:** RNA N^6^-methyladenosine, immunoregulatory cells, bone homeostasis, rheumatoid arthritis, epigenetics

## Abstract

Rheumatoid arthritis (RA) is a systemic autoimmune disease for which the etiology has not been fully elucidated. Previous studies have shown that the development of RA has genetic and epigenetic components. As one of the most highly abundant RNA modifications, the N^6^-methyladenosine (m^6^A) modification is necessary for the biogenesis and functioning of RNA, and modification aberrancies are associated with various diseases. However, the specific functions of m^6^A in the cellular processes of RA remain unclear. Recent studies have revealed the relationship between m^6^A modification and immune cells associated with RA. Therefore, in this review, we focused on discussing the functions of m^6^A modification in the regulation of immune cells and immune-related bone homeostasis associated with RA. In addition, to gain a better understanding of the progress in this field of study and provide the proper direction and suggestions for further study, clinical application studies of m^6^A modification were also summarized.

## Introduction

Rheumatoid arthritis (RA) is a systemic autoimmune disease characterized by inflammatory changes in the joints synovial tissue, bone and cartilage of joints, with changes in extra-articular sites occurring less frequently (Scherer et al., 2020), substantially burdening both individuals and society (Cross et al., 2014). The etiology of RA has not been fully elucidated, but is thought to be associated with autoimmunity, infection, and provoking environmental factors. Recently, it has become evident that RA can occur due to genetic and epigenetic components. Previous genealogical studies and modern molecular-genetic investigations have also confirmed the involvement of genetic factors in the development of RA (Sparks and Costenbader, 2014). However, genetic heterogeneity does not appear to explain all the features of RA. Thus, investigations of epigenetic factors and mechanisms correlated with RA progression and response to treatment are increasingly significant (Glant et al., 2014; McGee and Hargreaves, 2019).

Epigenetics is the study of heritable changes in gene expression that do not involve alterations in the DNA/RNA sequence, including DNA methylation, histone, and RNA modifications (Arguello et al., 2019; McGee and Hargreaves, 2019). First reported in the 1970s, the reversible methylation of N^6^-methyladenosine (m^6^A) is the most prevalent internal messenger RNA (mRNA) modification in eukaryotes including mammals, plants, *Drosophila* and yeast, as well as in viruses with a nuclear phase (Fu et al., 2014). This modification is installed by N^6^-adenosine methyltransferase. A 70 kD S-adenosyl-L-methionine (SAM)-binding subunit, also called MT-A70 (METTL3), was identified as one component of the m^6^A methyltransferase complexes in mammalian cells. Recent studies have characterized this complex, which comprises METTL3, METTL14 and Wilms tumor 1 associated protein (WTAP). METTL14 and METTL3 are two active methyltransferases that form a heterodimer to catalyze m^6^A RNA methylation, while WTAP interacts with this complex and substantially affects mRNA methylation inside cells but not *in vitr*o. Knockdowns of these methyltransferases affect mouse embryonic stem cell differentiation (Fu et al., 2014). Since 2011, two m^6^A RNA demethylases of fat mass and obesity-associated (FTO) protein and AlkB homolog 5 (ALKBH5) have been discovered that are involved in mammalian development, RNA metabolism and fertility (Jia et al., 2011; Fu et al., 2014). These findings reveal the first examples that RNA modification is reversible and indicate their regulatory effects on mRNA and specific non-coding RNAs that contain m^6^A (Coker et al., 2019). Subsequent profiling of m^6^A distributions in mammalian transcriptomes and the recent mapping of the yeast m^6^A methylome in the meiotic state further confirm the dynamic nature of m^6^A modification (Cao et al., 2016). In addition, various studies have shown that m^6^A is preferentially centered around stop codon and at 3′untranslated regions (3′UTRs), as well as in long internal exons and at transcription start sites (TSSs). RNA-binding proteins has been shown to be affected by m^6^A, such as heterogeneous nuclear ribonucleoprotein G (HNRNPG). HNRNPG is a new m^6^A reader protein that can recognize a motif exposed by m^6^A modification through a low-complexity region (Liu et al., 2017). Furthermore, the protein human YTH domain family 2 (YTHDF2) was recently demonstrated to specifically recognize m^6^A-methylated mRNA and accelerate the decay of the bound mRNA (Chen et al., 2018). The results of these studies indicate that chemical modifications of m^6^A are common and important in a variety of biological processes.

Recent studies have demonstrated m^6^A modification is necessary for the biogenesis and functions of RNA, and modification aberrancies have been associated with various pathological process, such as obesity, systemic lupus erythematosus, and carcinogenesis (Wang et al., 2017; Wei et al., 2017; Li L. et al., 2018). In addition, m^6^A modification has been recognized as crucial regulator in the immune response and in immune cells. Thus, selectively altered m^6^A levels along with other types of immunotherapies may be effective management strategies in various of immunological diseases, such as RA. In this review, we focus on discussing the functions of m^6^A modification in regulating the innate and adaptive immune response, especially in immune cells and immune-related bone homeostasis associated with RA. In addition, we also summarize clinical application studies of m^6^A modification to gain a better understanding of the progress in this area and provide proper direction and suggestions for its further study.

## m^6^A Writers, Erasers, and Readers

**Figure 1:**
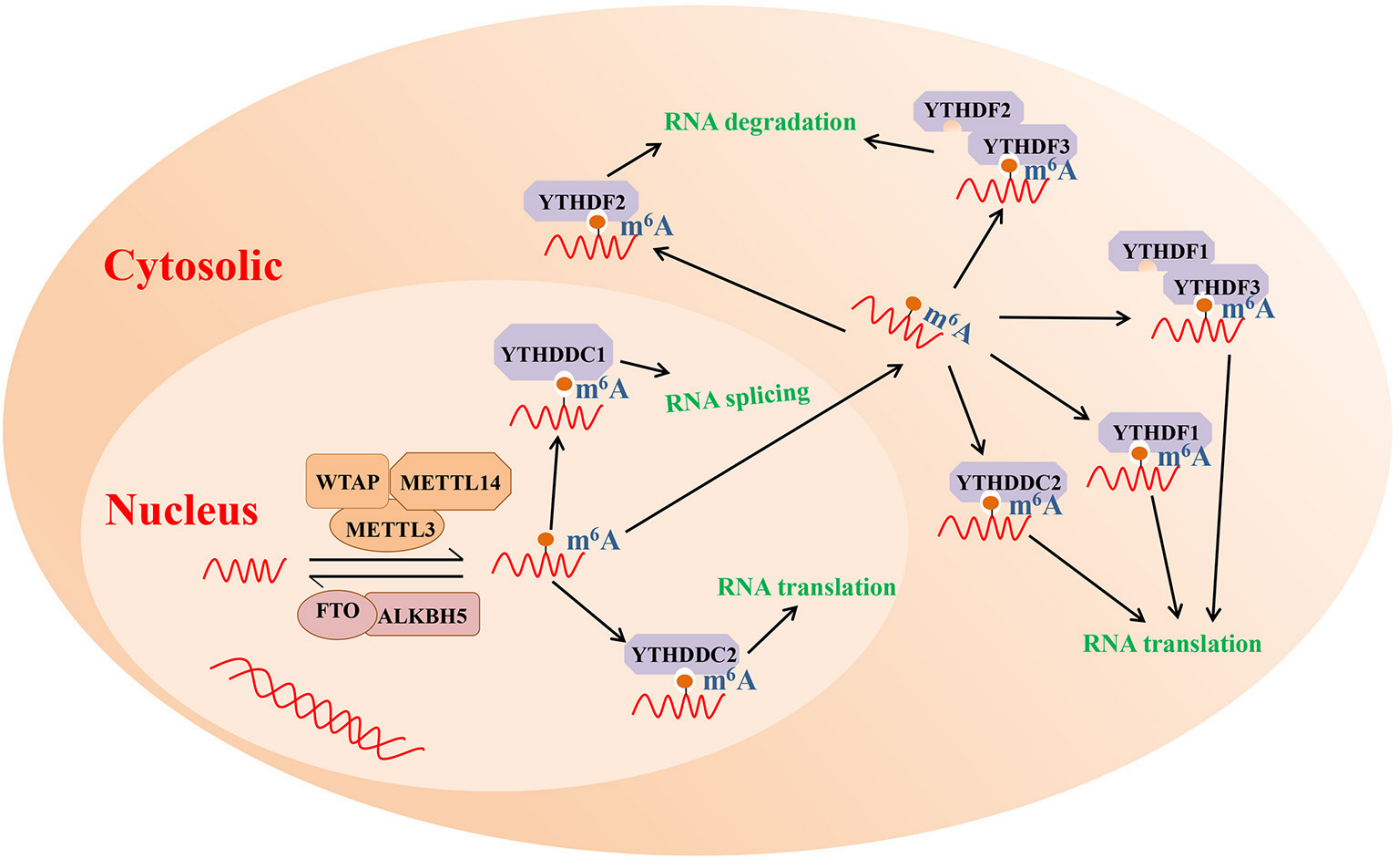
The potential molecular functions of m^6^A RNA modification. m^6^A modification is a reversible process mediated by its regulatory proteins, including the writers (MELTT3, METTL14, WTAP, etc.), erasers (FTO, ALKBH5, etc.), and readers (YTHDF1, YTHDF2, YTHDF3, etc.). ALKBH5, Alk B homolog 5; FTO, fat mass and obesity-associated protein; m^6^A, N^6^-methyladenosine; METTL3, methyltransferase like 3; METTL14, methyltransferase like 14; WTAP, Wilms tumor 1 associated protein; YTHDF1, YTH domain family 1; YTHDF2, YTH domain family 2; YTHDF3, YTH domain family 3.

As mentioned above, m^6^A modification is one of the most highly abundant RNA modifications and is involved in the etiology of various immune diseases, such as systemic lupus erythematosus and RA (Wei et al., 2017; Li L. et al., 2018). The results of numerous studies have revealed that m^6^A modification commonly occurs at the consensus motif RRACH (R = A or G; H = A, C or U) (Roundtree and He, 2016). Furthermore, m^6^A modification can interfere with RNA processing, splicing, export, degradation, and translation through the methyltransferase (m^6^A writers), demethylase enzymes (m^6^A erasers), and readers proteins (Chen et al., 2019). The potential molecular functions of m^6^A RNA modification are presented in Figure 1.

### m^6^A Writers

m^6^A mRNA methylation is catalyzed by a multisubunit writer complex' comprising a METTL3-METTL14 heterodimer and many additional adaptor proteins (Shulman and Stern-Ginossar, 2020). METTL3 was earlier identified as a SAM-binding component of “writer complex” and exhibited catalytic function by itself (Bokar et al., 1997). METTL3 knockdown can reduce m^6^A peaks and promote the apoptosis of HeLa and HepG2 cells (Dominissini et al., 2012; Liu et al., 2014). METTL3 located in nucleus and cytoplasm were observed, which is consistent with early findings showing that cytosolic extracts also possessed methyltransferase activity (Harper et al., 1990). As an allosteric activator, METTL14 forms a stable hetero complex with METTL3 and binds to the target RNA. Liu et al. showed that METTL14 knockdown decreased m^6^A levels in HeLa and 293FT cells (Liu et al., 2014). The other known writer complex also includes the WTAP, ZC3H13, RBM15 or RBM15B, and VIRMA subunits. WTAP is a splicing factor that can binds to the METTL3-METTL14 heterodimer and regulates the deposition of m^6^A inside cells (Liu et al., 2014). ZC3H13 maintains the nuclear localization of the complex, and RBM15/15B and VIRMA are regarded as providing additional specificity (Patil et al., 2016; Knuckles et al., 2018; Yue et al., 2018).

Except for the canonical writer complex, several other enzymes have been shown to act as methyltransferases of m^6^A, such as METTL16, MAT2A, ZCCHC4, METTL15 and PCIF1 (Pendleton et al., 2017; Akichika et al., 2019; Boulias et al., 2019; Ma et al., 2019; Sun et al., 2019; van Tran et al., 2019). Because the m^6^A modified positions generated by these enzymes have not been shown to be associated with immunity, this review focuses on the canonical writer complex of m^6^A mRNA modification.

### m^6^A Erasers

Two enzymes, FTO and ALKBH5, have been suggested to remove m^6^A from mRNA, indicating that m^6^A is a dynamically reversible modification of RNA. As the first identified RNA demethylase, FTO was discovered in 2011 and was important in reigniting investigations of m^6^A (Jia et al., 2011). FTO is a member of the non-heme Fe^II^/α-KG-dependent dioxygenase AlkB family of proteins, which also includes ABH1 to ABH8 (Kurowski et al., 2003; Gerken et al., 2007), and previous research revealed that FTO probably demethylates both m^6^A and terminal m^6^Am (Mauer et al., 2017; Wei et al., 2018). ALKBH5 was the second demethylase identified and specifically demethylates m^6^A (Zheng et al., 2013). Although m^6^A erasers allow for the dynamic and signal-dependent regulation of m^6^A levels, various studies have demonstrated that FTO and ALKBH5 has different intracellular localization patterns and tissue specificity. FTO is highly expressed in cerebral and adipose tissue, while ALKBH5 is highly abundant in the testes (Zheng et al., 2013). Thus, demethylation that occurs in some tissues may be regulated only by FTO or ALKBH5, indicating that the potential effects of RNA demethylation in more physiological contexts need to be further studied.

### m^6^A Readers

The m^6^A methylation or demethylation modification of target RNA occurs under the action of “writers” and “erasers,” respectively, altering the secondary or tertiary structure of RNA. The m^6^A-binding protein referred to as an m^6^A “reader” recognizes and preferentially binds to the m^6^A-modified RNA. For m^6^A modifications to exerts biological function, RNAs with m^6^A modifications need to be recognized by different “readers” to exert different downstream effects.

Members of the YT521-b homology (YTH) family are the primary “readers” that identified to date. In mammals, YTH domain-containing proteins that bind to m^6^A-modified RNAs are divided into five classes as follows. YTHDF1 interacts with initiation factors and promotes the binding of target RNAs to ribosomes, enhancing mRNA translation and protein synthesis (Zhuang et al., 2019). YTHDF2 selectively binds m^6^A-modified mRNA and then recruits it to the mRNA attenuation site to induce the degradation of transcription products. For example, (Du et al., 2016; Park et al., 2019), YTHDF2 triggers the adenylation and decay of m^6^A-modified mRNA by binding to the SH domain of the CNOT1 subunit in its N-terminal region, promoting its recruitment to the adenylase complex. Alternatively, YTHDF2 forms a complex with HRSP12 and RNaseP/MRP to mediate the cleavage of the m^6^A-modified RNA in the nucleus. Interestingly, YTHDF3 has been reported to have bidirectional activity, enhancing RNA translation when interacting with YTHDF1, while binding to YTHDF2 can promote RNA degradation (Shi et al., 2017). These three YTHDF family member proteins are primarily localized to the cytosol and have paralogs that are highly similar. YTHDC1 facilitates mRNA splicing (Xiao et al., 2016) and mRNA production in the nucleus (Roundtree et al., 2017). YTHDC2, which exhibits both cytosolic and nuclear expression, can promote the translation efficiency of target RNA (Mao et al., 2019). In addition to YTH readers, several other binding proteins have shown to bind to m^6^A-modified RNA, such as IGF2BPs, HNRNPA2B1, and EIF3 (Alarcón et al., 2015; Meyer et al., 2015; Huang et al., 2018).

## m^6^A Detection Methods

At present, numerous technologies involved in quantifying m^6^A levels and m^6^A modified transcripts have been developed to understand the phenotypes caused by the deletion of proteins associated with m^6^A modification and how m^6^A-modified transcripts are affected at the molecular level. According to their detection performance, these technologies can be classified as semiquantitative, quantitative, and localization-detecting methods (Chen et al., 2019).

Semiquantitative detection methods include dot blotting, immuno-Northern blotting and assay exploiting the methylation-specific sensitivity of MazF RNA endonucleases, which are applied to detect the presence of m^6^A modification. Dot blots are used to measure the global change in m^6^A levels using antibodies that specifically bind to the m^6^A site. Although this method has the advantages of being simple and fast, when m^6^A RNA fragments are small, its sensitivity is low (Zhu et al., 2019). Immuno-northern blotting is used for various types of RNA as it does not require RNA fragmentation prior to analysis. Thus, immuno-northern blotting is another technique used for the semi-quantitative detection of m^6^A modifications. This method is characterized by its high specificity, sensitivity, and quantitative capability (Mishima et al., 2015). MAZTER-seq takes advantage of the ability of the MazF RNase to cleave RNA at unmethylated ACA motifs but not at their methylated counterparts. This method was the first to provide systematic quantitative profiling of m^6^A at single-nucleotide resolution, but it only detects approximately 16–25% of mammalian m^6^A-modified sites due to its specificity (Garcia-Campos et al., 2019; Zhang Z. et al., 2019).

Unlike semi-quantitative methods, quantitative detection strategies include the electrochemical immunosensor method, photo-crosslink-based quantitative proteomics, and support vector machine-based method, which can be used to determine the levels of m^6^A RNA. In the electrochemical immunosensor approach, an anti-m^6^A antibody is used to recognize and capture the m^6^A-5′-triphosphate. Subsequently, silver nanoparticles and amine-PEG3-biotin functionalized SiO_2_ nanospheres (Ag@SiO_2_) are used to amplify the signal, after which phos-tag-biotin has the crucial function of connecting m^6^ATP and Ag@SiO_2_. This method is inexpensive, simple, and highly specific and sensitive (Yin et al., 2017). Photo-crosslinkers are widely used to stabilize protein-RNA interactions and can be combined with quantitative proteomics to measure m^6^A RNA levels. In addition, diazirine-containing RNA probes have also been synthesized and used to improve the efficiency of photo-crosslinkers (Arguello et al., 2017). The support vector machine-based method is a computational method proposed by Chen et al. to predict m^6^A sites within RNA strands using high-throughput sequencing data, a method is suitable for identifying m^6^A sites in plants (Chen et al., 2016).

At present, researchers have developed several methods to detect the specific locations of m^6^A sites within RNA. In combination with RNA immunoprecipitation (IP) whole-transcriptome sequencing, m^6^A levels and isoform-characterization sequencing (m^6^A-LAIC-seq) was invented by Molinie et al. to quantify m^6^A contents. However, although this method can be used to detect the m^6^A levels in each gene, it cannot analyze the methylation of a single modified nucleotide (Molinie et al., 2016). The use of m^6^A-seq provided the first global view of m^6^A-modified transcripts through transcriptome-wide sequencing and comparing m^6^A-antibody enriched regions to the input. This method has been widely used, promoting the identification of modified transcripts and demonstrating that m^6^A is a dynamically reversible modification enriched near stop codons and in 3′-UTRs (Dominissini et al., 2012; Meyer et al., 2012). However, the m^6^A-seq method can only localize m^6^A sites in RNA in 100–200 nt long regions and lacks single-base-resolution because of non-specific antibody binding. m^6^A-CLIP/IP and miCLIP are additional ultraviolet crosslinking strategies that can be used to determine the m^6^A site at a nucleotide-specific level and provide single-base resolution (Ke et al., 2015; Linder et al., 2015; Grozhik et al., 2017). In addition to the aforementioned methods, HRM analysis is another simple technique that can be used for m^6^A specific site detection as well as to screen knockout/knockdown strain libraries to identify genes contributing to the formation of a specific m^6^A nucleoside (Zhu et al., 2019).

## m^6^A Modification in Cells Involved in Ra

As an autoimmune disease, RA is characterized by the infiltration of multiple type of immune cells and their secreted inflammatory mediators in synovial tissue (Feldmann et al., 1996). Both innate and adaptive immune cells are involved in the pathogenesis of RA. Many studies have shown that macrophages cannot only engulf and kill pathogenic microorganisms but also secrete a variety of inflammatory factors to participate in the pathogenic process of RA. Furthermore, during the pathogenesis of RA, a variety of factors disrupt the equilibrium of M1/M2 macrophages, resulting in a skew toward M1-pro-inflammatory macrophages (Fukui et al., 2017; Sun et al., 2017; Siouti and Andreakos, 2019). In addition, a large number of dendritic cells (DCs) migrate to synovial tissues and fluids and highly express MHC and co-stimulators in patients with RA (Thomas et al., 1994). Macrophages and DCs act as specialized antigen presenting cells (APCs) to stimulate the T cell response (Mulherin et al., 1996; Leung et al., 2002; Merad et al., 2013). In RA patients, abnormal T cell activation is involved in the onset of RA and greatly contributes to joint destruction. Thus, the homeostasis of immune cells is crucial to maintaining health. Moreover, in the pathological condition of RA, bone homeostasis, involving bone formation mediated by osteoblasts and bone resorption regulated by osteoclasts, is disrupted. Due to the important role of m^6^A modifications in immune regulation, below we have summarized the dramatical effects of m^6^A modifications in different immunoregulatory cells and on bone homeostasis associated with RA.

### m^6^A Modification in Peripheral Blood Mononuclear Cells (PBMCs)

Accumulating evidence have shown that immune system dysfunctions, such as the abnormal activation of T cells, B lymphocytes, mast cells, neutrophils and macrophages contributes to promoting the onset of RA and is involved in the mechanisms of RA (Cascão et al., 2010; Klareskog et al., 2013). Table 1 lists a number of studies describing m^6^A modifications in PBMCs associated with RA that involved m^6^A writers, erasers and readers. Luo et al. assessed ALKBH5, FTO and YTHDF2 mRNA expression in PBMCs from RA patients using quantitative real-time polymerase chain reaction, with the global m^6^A content measured using an m^6^A RNA methylation quantification kit. The results showed that the mRNA expression of ALKBH5, FTO and YTHDF2 was lower in PBMCs from RA patients than in controls. After standard treatment, ALKBH5 mRNA levels were increased in RA patients. In addition, FTO expression is associated with some common markers for RA disease activity, including disease activity score 28 (DAS28), immunoglobulin G (IgG), complement 3 (C3), and the lymphocyte-to-monocyte ratio (LMR). YTHDF2 mRNA expression was shown to be correlated with L%, N%, RBC, NLR, and LMR. Moreover, logistic regression analysis suggested that reduced ALKBH5, FTO, and YTHDF2 expression in PBMCs is a risk factor for RA (Luo et al., 2020). Another study revealed that METTL3 expression is significantly increased in the PBMCs of RA patients (Wang J. et al., 2019). As a long-term autoimmune disease of unknown etiology, it is crucial to identify a biomarker signature that can predict joint damage at an early stage, which will support more informed clinical decisions on the most appropriate treatment strategies for individual patients. Although the exact mechanisms by which m^6^A modifications in PBMCs affect RA have not been fully elucidated, the results of the above studies suggested that the “writers,” “erasers” and “readers” of m^6^A modifications may serve as biomarkers for the diagnosis of RA.

**Table 1:** m^6^A modification in PBMCs of RA patients.

**m^**6**^A component**	**Regulation**	**Samples and conditions**	**References**
ALKBH5, FTO, YTHDF2	Down regulation	79 RA patients and 61 healthy controls	Luo et al., 2020
METTL3	Up regulation	47 RA patients and 30 healthy controls	(Wang J. et al., 2019)

*AKBH5, Alk B homolog 5; FTO, fat mass and obesity-associated protein; METTL3, methyltransferase-like 3; RA, rheumatoid arthritis; YTHDF2, YTH domain family 2*.

### m^6^A-Mediated Regulation of Macrophage Activation and Polarization

Macrophages are a subset of mononuclear phagocytic cells. During the RA progression, macrophages play distinct roles in the innate immunity function that causes inflammation. Activated macrophages are classified as two types and exhibit different polarization states based on the presence of pathogens and the expression of cytokines in the microenvironment. M1 macrophages, or classically activated macrophages, secret principally proinflammatory cytokines such as tumor necrosis factor (TNF)-α and interleukin (IL)-1 and cause joint erosion. M2 macrophages, or alternatively activated macrophages, can produce anti-inflammatory cytokines mainly including transforming growth factor (TGF)-β and IL-10 contributing to vasculogenesis, tissue remodeling and repair (Kinne et al., 2000). Mechanistically, macrophage polarization is regulated by a variety of signaling molecules and associated pathways, such as nuclear factor kappa-B (NF-κB) and the janus kinase/signal transducer and activator of transcription (JAK/STAT) signaling pathway, which are all associated with RA (Kubo et al., 2018; Li H. et al., 2018). Recently, m^6^A modification has been shown to alter the mRNA levels of specific signaling molecules and affect the polarization state of macrophages. *In vitro*, macrophages are typically activated by pathogenic factors, leading to the phosphorylation and translocation of the transcription factor NF-κB. When METTL3 is overexpressed, the nuclear translocation of phosphorylated NF-κB in cells is inhibited. Wang et al. found that METTL3 inhibits the activation of phor-bol 12-myristate 13-acetate (PMA)-induced macrophage-like cells (pTHP-1 cells) and attenuates the lipopolysaccharide (LPS)-induced inflammatory response in pTHP-1 macrophages through the NF-κB signaling pathway (Wang J. et al., 2019). METTL3-mediated m^6^A modification also facilitates the expression of STAT1 by enhancing mRNA stability, thereby altering the polarization type of macrophages. Moreover, METTL3 silencing significantly suppresses IFNγ-induced M1 macrophage polarization while promoting the expression of M2 macrophage marker genes (Liu Y. et al., 2019), indicating METTL3 potentially serving as an anti-inflammatory target in RA.

As an m^6^A erasers, FTO affects both the phosphorylation of NF-κB and the expression of STAT1 during macrophage polarization. Following FTO depletion, both the phosphorylation levels of several elements in the NF-κB signaling pathway and the mRNA stability of STAT are decreased, resulting in macrophage activation being blocked (Gu et al., 2020). As an m^6^A-binding protein, YTHDF2 may be involved in the above processes. The primary function of YTHDF2 is to selectively bind to m^6^A-modified mRNA and recruit it to the mRNA decay site to induce transcription product degradation. Furthermore, YTHDF2 participates in regulating the LPS-stimulated macrophage inflammatory response. Yu et al. found that YTHDF2 knockdown could increase the expression and stability of MAP2K4 and MAP4K4 mRNA through stabilizing the mRNA transcripts, which activate MAPK and NF-κB signaling pathways, promote proinflammatory cytokines expression and aggravate the inflammatory response in LPS-induced RAW264.7 macrophages cells. (Yu et al., 2019). In view of the important role of macrophages in RA, although there was no study about m^6^A in macrophages related to RA directly such as macrophages from collagen-induced arthritis model, these findings *in vitro* provide new directions for epigenetic studies in the pathogenesis of RA.

### m^6^A-Mediated Regulation of Dendritic Cell Activation and Function

**Figure 2:**
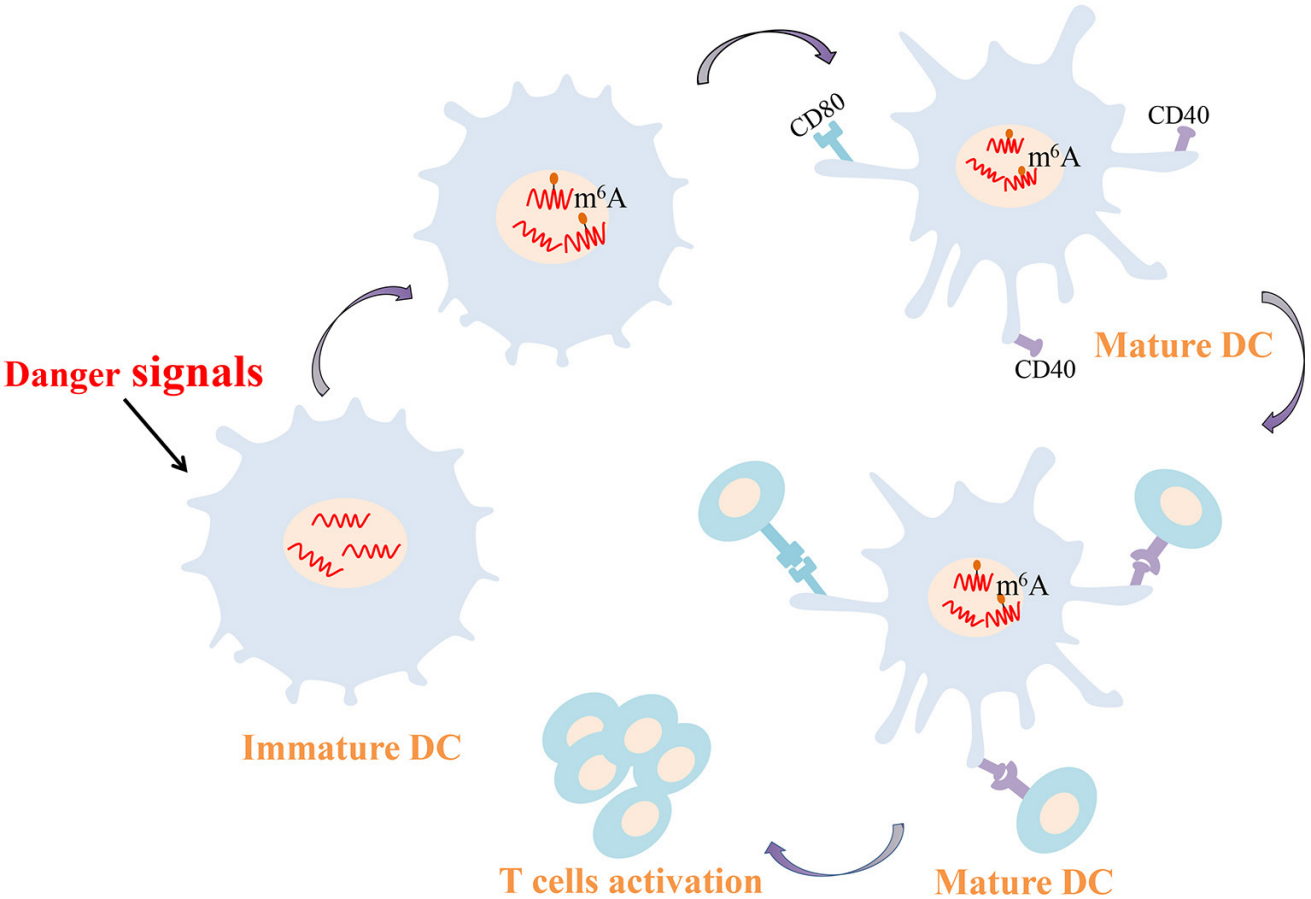
m^6^A-mediated regulation of dendritic cell. m^6^A modification increases the expression of CD40 and CD80 conducive to the capacity of DCs that presents antigens and stimulates T cells. DCs, dendritic cells; m^6^A, N^6^-methyladenosine.

As professional antigen presenting cells, DCs connect the innate and adaptive immune responses. Activated DCs have the striking ability to capture and present antigens and can express major histocompatibility complexes and co-stimulating molecules to activate T cells. DCs and T cells interactions consolidate the generation of an autoimmune response in RA. In RA patients, DCs are recruited to joint synovial fluid and tissues in high concentration, and synovial DCs are generally mature and NF-κB is overexpression (Cheung and McInnes, 2017; Wehr et al., 2019). Furthermore, compared with healthy controls DCs, CD1c^+^ DCs from RA secrete increased amounts of proinflammatory cytokines such as IL-1β, IL-6, IL-12 and IL-23, which are crucial players in the pathogenesis of RA (Lebre et al., 2008). Recent study confirmed that METTL3-mediated m^6^A modification increases the expression of CD40, CD80, and Tirap mRNA both *in vivo* and *in vitro*. The up-regulated CD40 and CD80 are conducive to the capacity of DCs that presents antigens and stimulate T cells (Figure 2). Higher Tirap expression enhances Toll-like receptor (TLR4)/NF-κB signaling pathway in DCs and increase the secretion of IL-6 and IL-12 (Wang H. et al., 2019). CC-chemokine receptor 7 (CCR7) stimulation is well known to induce DCs to migrate into lymph nodes in a unique manner, which is essential for initiating protective immunity and maintaining immune homeostasis (Förster et al., 2008; Seth et al., 2011). A long non-coding RNA lnc-Dpf3 has been reported to be involved in this mechanism (Liu J. et al., 2019). Following CCR7 stimulation, m^6^A modification of lnc-Dpf3 is down-regulated. Moreover, m^6^A can be recognized on lnc-Dpf3 by YTHDF2, but not YTHDF1. CCR7 stimulation down-regulates the recognition of lnc-Dpf3 by YTHDF2 and then reduces its degradation. The two effects described above increase the expression of lnc-Dpf3, which then interacts with the transcription factor hypoxia inducible factor 1-α (HIF-1α) to inhibit the HIF-1α-dependent glycolysis. HIF-1α-dependent glycolysis is one of the metabolic pathways that promotes maturation and migration of DCs Which ultimately prevents the amplification of inflammatory responses (Guak et al., 2018; Liu J. et al., 2019). At present, the study about m^6^A modification in synovial DCs from RA is rare, but the above findings may provide new ideas for epigenetic researches of RA in the future.

### m^6^A Regulation of T Cell Homeostasis

**Figure 3:**
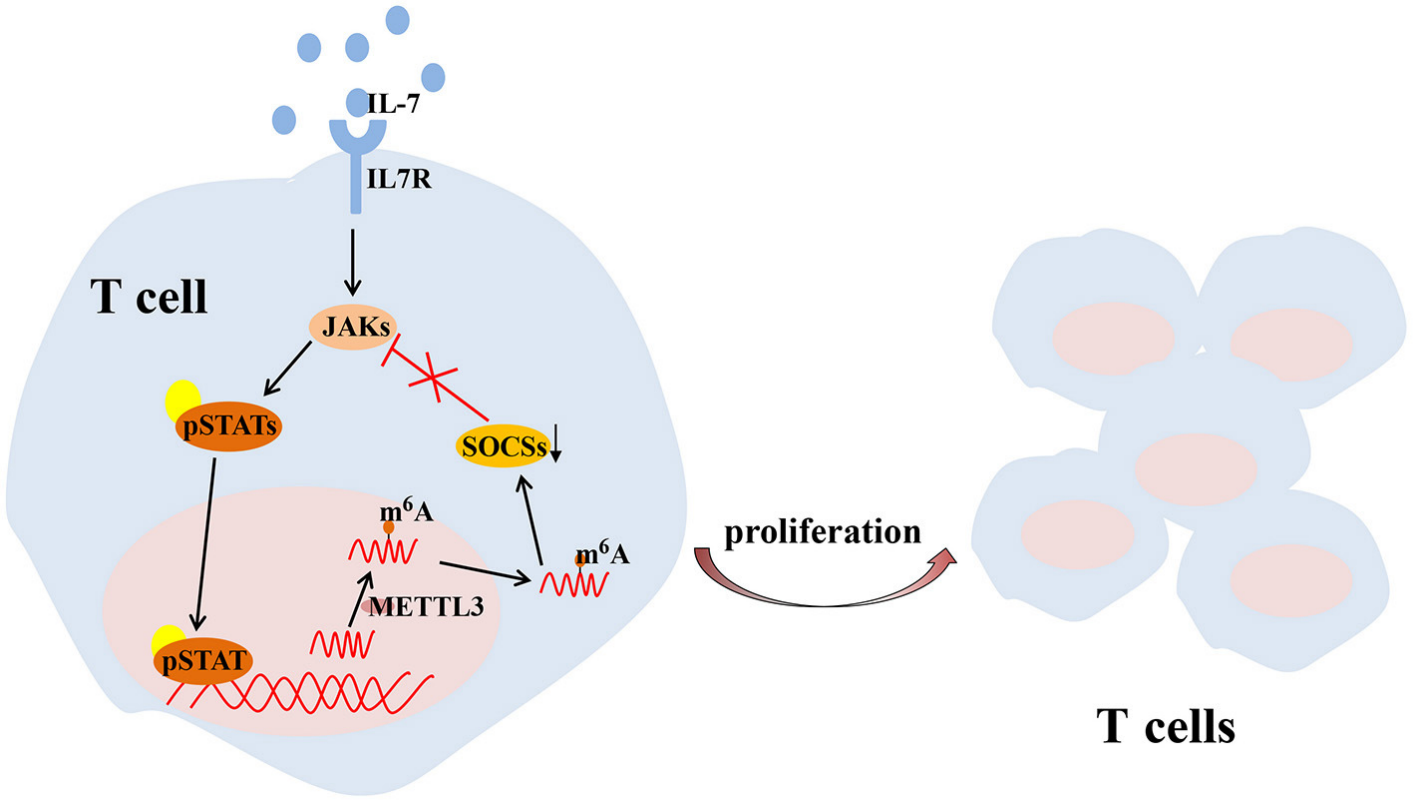
METTL3-mediated m^6^A mRNA methylation controls T cell proliferation by targeting IL-7/STAT/SOCS pathway. IL-7, interleukin 7; m^6^A, N^6^-methyladenosin; METTL3, methyltransferase like 3; SOCS, suppressor of cytokine signaling; STAT, signal transducer and activator of transcription.

T cell is one of the key regulators of synovial inflammation and recognized as a contributor to the progressive joint destruction that is a hallmark of RA, which has both stimulatory and inhibitory effects and plays a destructive or a protective role in bone metabolism in a context-and subtype-dependent manner (McInnes and Schett, 2011). Several studies have shown that mRNA methylation due to m^6^A modification is crucial for maintaining T cell homeostasis, which is essential to maintain the size of the T cell pool and lay the foundation for adaptive immunity. Physiologically, members of the suppressor of cytokine signaling (SOCS) protein family are important inhibitors of the JAK-STAT signaling pathway, including SOCS1, SOCS3, and CISH. SOCS1 is a significant negative regulator of IL-7, while SOCS3 and CISH inhibit STAT5 phosphorylation and T cell proliferation (Yoshimura et al., 2007; Palmer and Restifo, 2009). METTL3 has been reported to affect the stability and expression of SOCS1, SOCS3 and CISH mRNA in cells to regulate the balance between TCR-mediated ERK/AKT signaling and IL-7-mediated JAK-STAT signaling to control T cell homeostasis (Li et al., 2017; Furlan et al., 2019) (Figure 3).

T cells are complex lymphocytes, and there can be subpopulations of these cells with different developmental stages or functions. In RA, type 1 T helper (Th1)-cell and Th17-cell subsets have important pathogenic roles and are major contributors to synovial inflammation as well as to cartilage and bone destruction. And a disrupted balance between Th1 and Th2 has been thought to driven the pathology of organ in RA (Kobezda et al., 2014). Compared to wild-type naive T cells, fewer Th1 and Th17 cells and more Th2 cells are present during cell differentiation among naive T cells lacking METTL3, while Tregs remained unchanged. The specific absence of METTL3 in Tregs leads to the increased expression of SOCS, which inhibits the IL2-STAT5 signaling pathway (Li et al., 2017; Tong et al., 2018) an essential pathway for maintaining the immunosuppressive function of Tregs. It may be considered that m^6^A is an important regulatory factor for helper T cell differentiation and to maintain the functions of various T cells.

In addition, previous study revealed that autoimmune arthritis occurred with a wide spectrum among HIV-infected patients (Lawson and Walker-Bone, 2012). It had been observed that RA was worsening with antiretroviral therapy in the era of highly active antiretroviral therapy, which might reveal a pivotal role of CD4^+^ T lymphocytes in the pathogenesis of RA (Siva and Brasington, 2001). At present, m^6^A modification has been shown to be involved in the antiviral mechanism of T cells and hosts. Kennedy and Lichinchi et al. suggested that posttranscriptional m^6^A modification and the YTHDF1-3 protein are positive regulators of HIV-1 infection. The overexpression of YTHDF1-3 protein in CD4+ T cells increased viral replication as well as the expression of HIV-1 viral protein (Kennedy et al., 2016; Lichinchi et al., 2016). In contrast, Tirumuru et al. showed that YTHDF1-3 is an inhibitor and reduced HIV-1 reverse transcription to inhibit HIV-1 infection in CD4^+^ T cells after its overexpression (Tirumuru et al., 2016). This discrepancy may be due to the different detection methods or different roles of YTHDF1-3 in different cell lines. In summary, m^6^A methylation may indirectly regulate the antiviral effect of the host by regulating T cells. As T cells regulate the entire adaptive immune response and have crucial roles in RA, the above findings open new avenues of investigation into the function of m^6^A in RA and further suggest that T cell-specific delivery of m^6^A-modifying agents might be an effective treatment to alleviate RA.

### m^6^A Modification in Immune-Related Bone Homeostasis

Bone is composed of a highly calcified intercellular stroma, namely, bone matrix, and varieties of cells. Bones are in a constant state of transition from old to new. In the process of physiological reconstruction, osteogenesis and osteolysis are in a state of dynamic equilibrium. Osteogenesis promotes the growth, repair, and reconstruction of bone, which is primarily performed by osteoblasts. Under specific conditions, bone dissolves to release Ca^2+^, maintaining a constant level of blood calcium. The dissolution of bone is called osteolysis and is primarily performed by osteoclasts. Osteoclasts absorb bone tissues through enzymatic degradation and participate in bone reconstruction and blood calcium balance. RA is characterized by synovitis, with the infiltration numerous inflammatory cells. Due to the expression of proinflammatory cytokines in inflammatory synovial cells, osteoclast differentiation is promoted, while osteoblast function is inhibited. If this condition persists, the bone balance will be disrupted and then lead to the destruction of bone and articular cartilage (Karmakar et al., 2010; Baum and Gravallese, 2014; Schett, 2017).

**Figure 4:**
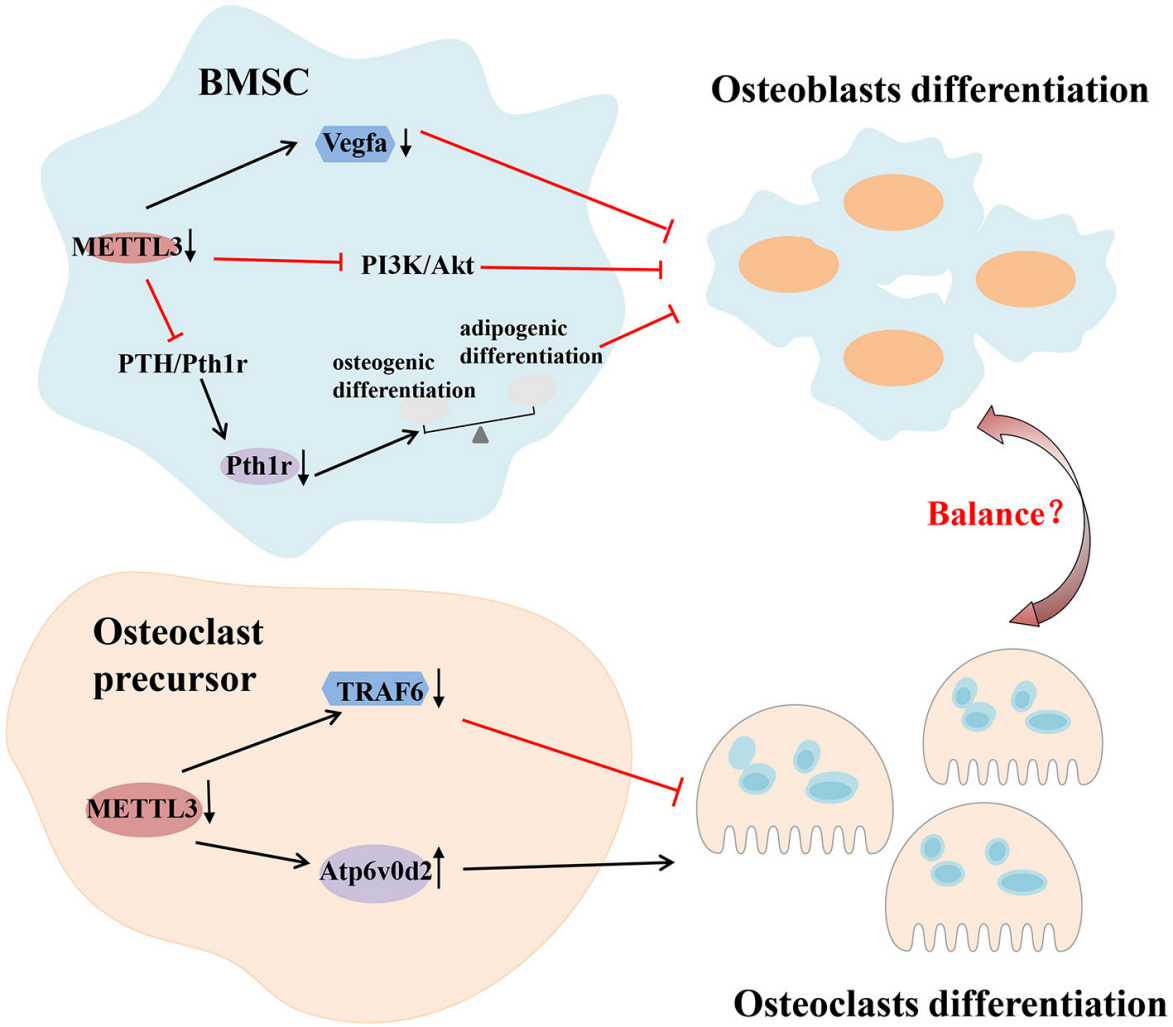
m^6^A modification in immune-related bone homeostasis. Deletion of METTL3 in BMSCs can inhibit osteoblast differentiation by inhibiting the PTH/Pth1r, PI3K/Akt signaling pathway and limiting the expression of vascular endothelial growth factor A (Vegfa). As for osteoclast, METTL3 regulates the differentiation and function of osteoclast through different mechanisms which involve Atp6v0d2 mRNA degradation mediated by YTHDF2 and Traf6 mRNA nuclear export. Akt, protein kinase B; Atp6v0d2, V-type proton ATPase subunit d 2; BMSCs, bone marrow mesenchymal stem cells; METTL3, methyltransferase like 3; PI3K, phosphoinositide-3-kinase; PTH, parathyroid hormone; Pth1r, parathyroid hormone 1 receptor; Traf6, tumor necrosis factor receptor-associated factor 6; YTHDF2, YTH domain family 2.

Several experiments have shown the importance of m^6^A modification in bone remodeling. Abnormal changes of m^6^A machinery proteins, including “writer,” “eraser” and “reader,” lead to the impaired differentiation and dysfunction of osteocytes. For example, the absence of METTL3 in bone marrow mesenchymal stem cells (BMSCs) reduces the expression of parathyroid hormone receptor-1 (Pth1r) in mammals by inhibiting the Parathyroid hormone (PTH)/Pth1r signaling pathway, thereby blocking the synthesis of parathyroid hormone. These deletions tilt parathyroid hormone-induced osteogenic differentiation toward adipogenic differentiation, leading to bone loss and excessive bone marrow fat (Wu et al., 2018). The deletion of METTL3 in BMSCs can also inhibit osteoblast differentiation by limiting the expression of vascular endothelial growth factor A (Vegfa) and its spliceosomes associated with bone formation (Tian et al., 2019). However, it has been reported that METTL3 overexpression *in vitro* significantly reduces the expression of osteogenic related genes (Mi et al., 2020). These results indicate that either too high or too low METTL3 expression may be detrimental to osteoblast differentiation. Osteoblast differentiation is also dependent on the regulation of FTO. Studies have shown that miR-22-3p and miR-149-3p modulate the osteoblast differentiation of BMSCs by targeting FTO (Li et al., 2019; Zhang et al., 2020). Mice with a global loss of FTO or a selective lack FTO in osteoblasts exhibit bone loss (Sachse et al., 2018; Zhang Q. et al., 2019). Li et al. established an osteoclastogenesis model using RANKL-induced RAW264.7 cells and observed that m^6^A level and METTL3 expression were increased during osteoclast differentiation. METTL3 knockdown resulted in an increased size of osteoclasts, but the bone-resorbing ability of osteoclasts was decreased. The mechanism associated with this process involved Atp6v0d2 mRNA degradation mediated by YTHDF2 and Traf6 mRNA nuclear export (Li et al., 2020). In addition, the abnormal differentiation and activation of osteoclasts can also cause bone homeostasis disorders, leading to various bone resorptive diseases. Knocking down METTL3 in preosteoclast expands the volume of mature osteoclasts, while the amount and the capacity of bone resorption are reduced (Li et al., 2020). Therefore, the stability of METTL3 is essential for physiological bone remodeling (Figure 4).

Inflammation is destructive to normal bone remodeling in RA. Due to the effect of m^6^A on bone remodeling, the unique role of m^6^A in the inflammatory environment on bone remodeling has been gradually recognized. In the inflammatory environment, METTL3 knockdown inhibits the transcription and protein levels of osteoblast factors, including Runx2, Sp7, Alpl, and Col1a1 while phosphorylation of the Smad1/5/9 complex is inhibited. Thus, insufficient Runx2 is recruited in the nucleus to activate osteogenic gene expression, resulting in the negative regulation of osteogenic differentiation (Zhang Y. et al., 2019). In another study, METTL3 silencing was shown to inhibit inflammatory responses and extracellular matrix (ECM) synthesis in chondrocytes treated with IL-1β, decreasing the chondrocyte apoptosis (Liu Q. et al., 2019). Rheumatic immune diseases eventually destroy the normal structure and function of bones, and the roles of methylation in immunity and bone remodeling has been demonstrated. Therefore, additional studies should be conducted to uncover the effects of m^6^A in RA.

### m^6^A Modification Regulates Cytokines and Cytokine receptors Signaling

Cytokines are small molecular polypeptides or glycoproteins that are primarily synthesized and secreted by immune cells. And cytokines can act on immune cells. Cytokines mediate intercellular interactions between cells and are involved in regulating the immune and inflammatory responses. Among them, inflammatory cytokines, including TNF-α, IL-6, TGF-β, and IL-10, have the closest association with the occurrence and development of RA (Feldmann et al., 1996). LPS is a recognized inducer of inflammatory responses. After adding LPS, increased mRNA levels of IL-6, IL-12, and TNF-α in preosteoblasts indicates a successful induction of inflammatory environment. Knocking down METTL3 can increase the expression of these inflammatory factors (Zhang Y. et al., 2019). In general, the combination of YTHDF2 and m^6^A modification leads to mRNA degradation, which can reduce the expression of proinflammatory cytokines. Thus, the deletion of YTHDF2 results in increased TNF-α, IL-1β, IL-6, and IL-12 expression in macrophages. Interestingly, YTHDF2 deletion does not directly affect the stability of these cytokines but rather dose so by promoting the phosphorylation of their upstream molecules p65, P38, and ERK (Yu et al., 2019). In the section on the m^6^A-mediated regulation of T cell homeostasis, we noted that SOCS protein family members inhibit IL-7 receptor signaling and negatively regulate T cell homeostasis proliferation. Not coincidentally, the degradation and expression level of SOCS mRNA can change in response to m^6^A markers (Li et al., 2017). In addition, the secretion of proinflammatory cytokines aids in controlling the inflammatory response influences the activity of bone cells, METTL3 knockdown can inhibits osteoblast differentiation via YTHDF2 involvement and activates the inflammatory response by regulating MAPK signaling in LPS-induced inflammation (Zhang Y. et al., 2019).

As an autoimmune disease, RA is related to the overexpression of proinflammatory cytokines and the abnormal up-regulation of cytokines is prevalent in RA patients (Gao et al., 2018). It has been proved that interferons (IFNs), acting as a subgroup of cytokines, can make crucial effects on regulating immunology, as well as modulating the dynamic balance of bone matrix. Type I IFNs consist of a multi-gene family including IFN-α and IFN-β. IFN-α has the ability to activate osteoblast differentiation and inhibits osteoclast fusion to maintain bone matrix integrity. Meanwhile, IFN-β exerts effects of suppressing osteoblast-mediated bone remodeling and inhibiting osteoclast differentiation to attenuate bone resorption (Deng et al., 2020). DEAD-box (DDX) helicases have been shown to demethylate m^6^A-modified transcripts to participate in type I IFNs activation by recruiting ALKBH5. This process enhances the preservation of transcripts in the nucleus to inhibit the production of type I IFNs, ultimately inhibiting the antiviral innate immune response (Zheng et al., 2017). However, another study where METTL3 was knocked down appears to have shown the opposite effect, indicating that m^6^A is a negative regulator of the type I IFNs response (Winkler et al., 2019). Do these two mechanisms work together to regulate type I IFNs production and further play a role in RA? Further studies are needed to elucidate these questions.

## m^6^A Modification in Clinical Application

As a refractory disease, it is essential to develop drugs that can target the molecules associated with RA. Novel RNA-targeted therapeutics, which are poised to offer high efficacy and specificity, provide inherent advantages and have shown convincing prospects in the treatment of various diseases (Dori and Soreq, 2006; Crooke et al., 2018; Scoles and Pulst, 2018; Tsimikas, 2018). The involvement of m^6^A-based medication in various immune and bone cells associated with RA has promoted extensive research efforts in m^6^A-based therapy. As previously mentioned, RNA processing, splicing, export, degradation, and translation can be interfered with by m^6^A modification through the activity of “writer,” “eraser,” and “reader” protein making it possible that developing inhibitors or promoters of “writers,” “erasers,” and “readers” to control the disease progression. In Table 2, we have summarized several inhibitors have been discovered via natural product analyses, chemical synthesis or biochemical- or cell-based small molecule compound library screening.

**Table 2:** Inhibitors and mechanisms of FTO discovered to date.

**Inhibitors' name**	**Experimental subject (*in vivo*/*in vitro*)**	**Mechanisms of inhibitors**	**References**
Rhein	BE (2)-C cells	Competitively binds to the FTO active site *in vitro*	Chen et al., 2012
Meclofenamic acid	HeLa cells	Competes with FTO on m^6^A-containing substrate binding over ALKBH5	Huang et al., 2015
N-CDPCB, 1a	3T3-L1 cells	An antiparallel β-sheet and the L1 loop of FTO sandwich N-CDPCB	He et al., 2015
CHTB	3T3-L1 cells	CHTB can bind to the FTO active site	Qiao et al., 2016
Entacapone	DIO mice/Hep-G2 cells	Directly bound to FTO and inhibited FTO activity *in vitro*	Peng et al., 2019
Radicicol	——	Adopts an L-shaped conformation in the FTO binding site and occupies the same position as N-CDPCB	Wang et al., 2018

*CHTB, 4-chloro-6-(6′-chloro-7′-hydroxy-2′,4′,4′-trimethyl-chroman-2′-yl)benzene-1,3-diol; DIO, diet-induced obese; FTO, fat mass and obesity associated protein; N-CDPCB, 1a, N-(5-Chloro-2,4-dihydroxyphenyl)-1-phenylcyclobutanecarboxamide; “——” indicates that there are no corresponding animals and cells used in the literature we cited*.

Until now, the target of inhibitors is almost FTO. In 2012, Chen and colleagues identified several small-molecule inhibitors of human FTO demethylase and observed that the natural product rhein could competitively bind to the FTO active site *in vitro* for the first time (Chen et al., 2012). Meclofenamic acid (MA) is a non-steroidal, anti-inflammatory drug that was identified as a highly selective inhibitor of FTO, which could compete with FTO for m^6^A-containing substrate binding over ALKBH5 (Huang et al., 2015). N-(5-Chloro-2,4-dihydroxyphenyl)-1-phenylcyclobutanecarboxamide (N-CDPCB, 1a) and 4-chloro-6-(6′-chloro-7′-hydroxy-2′,4′,4′-trimethyl-chroman-2′-yl) benzene-1,3-diol (CHTB) are the other inhibitors that bind FTO and were identified by virtual screening. The crystal structure of N-CDPCB, CHTB complexes with FTO show that N-CDPCB is sandwiched between an antiparallel β-sheet and the L1 loop of FTO as well as that CHTB can bind the FTO active site (He et al., 2015; Qiao et al., 2016). Furthermore, a previous study also observed that entacapone can directly bind to FTO and inhibit FTO activity *in vitro* (Peng et al., 2019). Radicicol, a nature compound, was also shown to be a potent inhibitor of FTO that can adopt an L-shaped conformation in the FTO binding site and occupy the same position as N-CDPCB (Wang et al., 2018).

Since METTL3 up-regulation and ALKBH5 and YTHDF2 down-regulation contribute to the progression of RA, METTL3, ALKBH5, and YTHDF2 may also be targeted for treatment. Indeed, Bedi et al. identified two series of adenine derivatives *in silico* by high-throughput docking of METTL3 and identified two compounds showing good ligand efficiency (Bedi et al., 2020). Although the inhibitors of METTL3, ALKBH5, and YTHDF2 are not available so far, these findings offer novel research directions.

## Discussion and Further Perspectives

**Figure 5:**
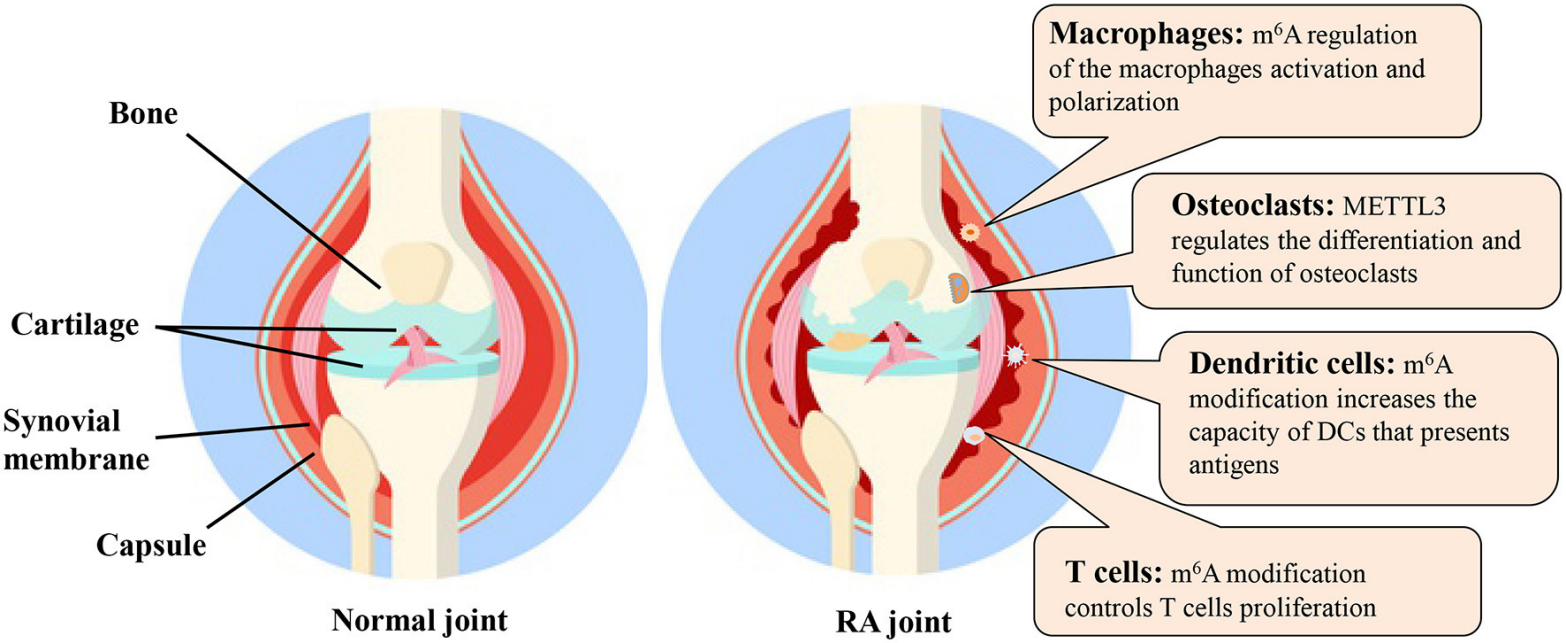
The m^6^A modification and possible molecular mechanisms in various cells related to RA. DC, dendritic cell; m^6^A, N^6^-methyladenosine; METTL3, methyltransferase like 3; RA, rheumatoid arthritis.

In this review, we briefly described the “writers,” “readers” and “erasers” of m^6^A and various detection methods for m^6^A modification. The m^6^A modification and relevant molecular mechanisms in various cells related to RA were also discussed (Figure 5). Although the field of m^6^A modification has become increasingly attractive, many research challenges and knowledge gaps remain.

Recently, analytical technology innovations have greatly promoted the study of m^6^A RNA modification. However, the selection and optimization of the laboratory technology m^6^A measurements in clinical practice remains an open problem. The application of these techniques in practical clinical m^6^A RNA is rare. Moreover, in addition to better detection sensitivity and accuracy, comprehensive detection and mapping the same biological sample with different modifications are desirable. Challenges pertaining to the needs of tools for deciphering the functions of m^6^A RNA modification will continue to stimulate the development of analytical methods and software.

The results of a growing number of preclinical studies have suggested that m^6^A modification is especially crucial in a variety of pathological and physiological conditions of immune and bone cells. We speculate that the potential primary effect of m^6^A modification in RA may be achieved through the regulation of different immune and bone cells. However, we observed that the percentage of m^6^A studies focused on RA is small. Mo et al. conducted a large-scale genome-wide association study to identify m^6^A-associated SNPs that affecting RA progression identifying 37 m^6^A-SNPs related to RA and 27 of them were verified to affect expression of 24 local genes in different RA cells or tissues (Mo et al., 2018). This result revealed the potential roles of m^6^A-SNPs in RA. Indeed, synovial hyperplasia is a hallmark in RA and is the primary contributor to the formation of an invasive pannus. In the progression of RA, synovial inflammation is dominated by the infiltration of immune cells into synovial tissue and by joint effusions rich in leukocytes compared to osteoarthritis (Scherer et al., 2020). Earlier studies of epigenetic changes in RA have demonstrated that differential DNA methylation genes can alter gene expression in fibroblast-like synoviocytes (FLS) and play a crucial role in RA pathogenesis (Karouzakis et al., 2009; Nakano et al., 2013). Thus, studies of m^6^A modification regarding synovial cells and tissues should be carried out in the future. In addition, as RA is a systemic and intricate disease, the mechanisms of m^6^A modification in RA require further investigation. Up to now, most of the conclusions in this field were based on the deletion of one of m^6^A machinery components. Therefore, determining how to bridge gaps between phenotype, specific methylated mRNAs and molecular mechanisms continues to be a major challenge. Furthermore, since the majority of essential components of m^6^A writers and erasers were deleted to conduct immunological phenotypes studies, a straightforward strategy is to identify the m^6^A readers such as YTHDF1 and YTHDF2, that can drive the observed phenotypes and determine whether the deletion of individual or multiple m^6^A readers can recapitulate these effects (Shulman and Stern-Ginossar, 2020). Recently, a study found that the YTHDF1-3, cytosolic m^6^A-binding proteins, could undergo liquid-liquid phase separation (LLPS) in cells. And this LLPS is markedly enhanced by mRNAs that contain multiple, but not single, m^6^A residues (Ries et al., 2019; Wang et al., 2020). Under the stress conditions, different RNAs and RNA-binding proteins form phase-separated, membraneless granules in cells. Stress granules (SGs) are RNA-protein granules that play crucial roles in epigenetic and post-transcriptional regulations. Fu et al. studied the localization of m^6^A-modified mRNAs and m^6^A-binding proteins, YTHDF proteins, in mammalians cells. They revealed that YTHDF proteins played an important role in SG formation (Fu and Zhuang, 2020). These findings may provide new ideas for m^6^A modification research.

In terms of the clinical application of m^6^A modification, the most important question may involve the development of drugs, as m^6^A-based drugs are currently scarce and poorly understood. There are only a limited number of inhibitors associated with FTO that have been identified, but their effects have only been experimentally verified, and an evaluation of their effectiveness and safety remains to be resolved. More importantly, there are no m^6^A-based drugs for RA at present. Thus, additional studies are needed to advance the application of m^6^A-based approaches for RA.

## Author Contributions

CX designed the framework of the review. DF, YX, and CL wrote the manuscript. QY and XX contributed in figure designing. QW and ZW contributed to the literature research. CW and CX revised and approved the manuscript. All authors contributed to the article and approved the submitted version.

## Conflict of Interest

The authors declare that the research was conducted in the absence of any commercial or financial relationships that could be construed as a potential conflict of interest.
